# Assessing Viral Shedding and Infectivity of Asymptomatic or Mildly Symptomatic Patients with COVID-19 in a Later Phase

**DOI:** 10.3390/jcm9092924

**Published:** 2020-09-10

**Authors:** Yujin Sohn, Su Jin Jeong, Won Suk Chung, Jong Hoon Hyun, Yae Jee Baek, Yunsuk Cho, Jung Ho Kim, Jin Young Ahn, Jun Yong Choi, Joon-Sup Yeom

**Affiliations:** 1 Division of Infectious Disease, Department of Internal Medicine, Severance Hospital, Yonsei University College of Medicine, 50-1 Yonsei-ro Seodaemun-gu, Seoul 03722, Korea; rubythyme@yuhs.ac (Y.S.); jsj@yuhs.ac (S.J.J.); ayu870213@yuhs.ac (J.H.H.); STELLANGELA.BAEK@yuhs.ac (Y.J.B.); YUNSUKC@yuhs.ac (Y.C.); qetu1111@yuhs.ac (J.H.K.); comebacktosea@yuhs.ac (J.Y.A.); seran@yuhs.ac (J.Y.C.); 2 College of Social Science, Yonsei University Graduate School, Seoul 03722, Korea; ssookhoppang@hanmail.net

**Keywords:** coronavirus disease 2019, COVID-19, RT-PCR, viral culture, saliva

## Abstract

Background: The coronavirus disease (COVID-19) pandemic, caused by severe acute respiratory syndrome coronavirus 2 (SARS-CoV-2), has become a major global public health issue. SARS-CoV-2 infection is confirmed by the detection of viral RNA using reverse transcription polymerase chain reaction (RT-PCR). Prolonged viral shedding has been reported in patients with SARS-CoV-2 infection, but the presence of viral RNA does not always correlate with infectivity. Therefore, the present study aimed to confirm the presence of viable virus in asymptomatic or mildly symptomatic patients in the later phase of the disease, more than two weeks after diagnosis. Method: Asymptomatic or mildly symptomatic COVID-19 patients who had been diagnosed with the disease at least two weeks previously and admitted to a community treatment center (CTC) from 15 March to 10 April 2020 were enrolled in this study. Nasopharyngeal and salivary swab specimens were collected from each patient. Using these specimens, RT-PCR assay and viral culture were performed. Result: In total, 48 patients were enrolled in this study. There were no significant differences in baseline characteristics between the asymptomatic and mildly symptomatic patient groups. RT-PCR assay and viral culture of SARS-CoV-2 were performed using nasopharyngeal and salivary swabs. The results of RT-PCR performed using salivary swab specimens, in terms of cycle threshold (Ct) values, were similar to those of RT-PCR using nasopharyngeal swab specimens. In addition, no viable virus could be cultured from swab specimens collected from the late-phase COVID-19 patients with prolonged viral RNA shedding. Conclusions: In conclusion, our study suggests that even if viral shedding is sustained in asymptomatic or mildly symptomatic patients with later phase of COVID-19, it can be expected that the transmission risk of the virus is low. In addition, saliva can be used as a reliable specimen for the diagnosis of SARS-CoV-2 infection.

## 1. Introduction

The coronavirus disease (COVID-19) pandemic, caused by severe acute respiratory syndrome coronavirus 2 (SARS-CoV-2), has become a major global public health issue. At the time of writing, the massive viral outbreak has affected 216 countries, with more than 13 million people infected and 580,000 having died [1].

In South Korea, the number of patients has increased rapidly since February 2020. For the proper management of COVID-19 patients, asymptomatic or mildly symptomatic patients were admitted to community treatment centers (CTCs), which are non-medical facilities for isolation and monitoring.

However, patients with prolonged viral RNA detection after resolution of symptoms presented difficulties in terms of their safe discharge from isolation. For efficient distribution of medical resources, the revised WHO discharge guidelines recommend that symptomatic patients should be discharged 10 days after the onset of symptoms, following at least three additional days without symptoms, and that asymptomatic patients should be discharged 10 days after testing positive for SARS-CoV-2 [2].

Diagnosis of COVID-19 is achieved using a nasopharyngeal swab. At present, real-time reverse transcription polymerase chain reaction (RT-PCR) analysis of respiratory specimens is the gold standard test for detecting SARS-CoV-2 infection [3].

Over the course of infection, viral RNA has been identified in respiratory tract specimens 1–3 days prior to the onset of symptoms. The viral load is thought to persist for up to 7–10 days after the onset of symptoms in mild cases. In more severe cases, it tends to peak up to 11 days after the onset, followed by gradual decrease over time [4]. Viral RNA has been detected in various specimens of the human body, such as the upper and lower respiratory tract, blood, pharyngeal swabs, saliva, urine, and feces [5,6].

Prolonged viral shedding has been reported from nasopharyngeal swabs up to 63 days after the onset of symptoms among adult patients [7,8]. However, viral shedding of SARS-CoV-2 does not always indicate infectivity, unless the virus is isolated and cultured from the specimens. The revised WHO release criteria do not require additional examination prior to discharge. Therefore, there is a residual risk that the disease can still spread when these criteria are adhered to. Accordingly, in late-phase COVID-19 patients with prolonged viral RNA detection, it is important to determine the risk of transmission.

In this study, we attempted to confirm the presence of viable virus by performing RT-PCR assay and culture using salivary and nasopharyngeal swabs of asymptomatic or mildly symptomatic COVID-19 patients who had been diagnosed with the disease and admitted to a CTC at least two weeks previously.

## 2. Methods

### 2.1. Patients and Study Settings

Asymptomatic or mildly symptomatic COVID-19 patients who had been admitted to the CTC between March 15 and April 10, 2020 and had been diagnosed at least two weeks previously, were enrolled in the current study. Asymptomatic or mildly symptomatic COVID-19 patients were defined as those with an early warning score of <3 for SARS-CoV-2 infection [9]. Patients referred to other hospitals due to worsening of symptoms during isolation were excluded. Written informed consent was obtained from all study participants. Nasopharyngeal and salivary swab specimens were obtained from each patient. Ethical Statement: This study was approved by the Institutional Review Board (IRB number: 4-2020-0133) of Severance Hospital (Seoul, South Korea) and informed consent was obtained.

### 2.2. Definitions

RT-PCR assays targeting three genes of SARS-CoV-2, the E (envelope protein), RdRP (RNA-dependent RNA polymerase), and N (nucleocapsid protein) genes, were performed using the Allplex™ 2019-nCoV Assay (Seegene Inc., Seoul, South Korea) with nasopharyngeal and salivary swab specimens. Patients with negative RT-PCR results were tested again by RT-PCR the following day; those with positive RT-PCR results were re-tested by RT-PCR after a week; and those with inconclusive RT-PCR results were re-tested by RT-PCR after three days. The inconclusive result refers to a case in which one or more, but not all genes included in the kit show an amplification curve after the cut-off when using a follow-up sample [10].

RT-PCR assay results were expressed as the cycle threshold (Ct) value. Ct values ≥ 40 were considered negative results. Negative conversion was defined as two consecutive negative RT-PCR results at a 24 h interval.

Rebound Ct value was defined as a negative (Ct value ≥ 40) from the single RT-PCR assay and positive (Ct value < 40) from the following RT-PCR result.

### 2.3. Nucleic Acid Extraction and RT-PCR for SARS-CoV-2

The nasopharyngeal swab sample was placed in 2 mL of viral transport medium. Saliva samples were collected in a sterile container and both specimens were stored frozen −70 °C. Nasopharyngeal swab specimens were subjected to total nucleic acid extraction using a viral RNA mini kit (QIAGEN, Hilden, Germany). After extraction, the total nucleic acid was recovered using 60 µL of elution buffer.

For salivary swab specimens, the same amount of PBS was added, and following the vortex process, RNA was extracted using the same process as for the nasopharyngeal swab samples. Using 8 µL of RNA, RT-PCR assay targeting the three genes of SARS-CoV-2 was performed using a Seegene Kit (Allplex 2019-nCoV Assay kit, Seegene, Korea). When the upper respiratory specimens including the nasopharyngeal swab were examined using the Allplex 2019-nCoV Assay, a Positive Percent Agreement (PPA) was 100% (95% CI: 92.75~100%), and a Negative Percent Agreement (NPA) was 93.07% (95% CI: 85.76~96.93%) [11].

### 2.4. SARS-CoV-2 Cell Culture

SARS-CoV-2 was cultured in a biosafety level 3 facility. Vero E6 cells were used for isolating SARS-CoV-2. Vero E6 cells were cultured in Eagle’s minimum essential medium (EMEM) supplemented with heat-inactivated fetal bovine serum (FBS; 10%). Both nasopharyngeal and salivary swab specimens were used for virus isolation. Vero E6 cells were seeded with 1 mL of EMEM at a density of 1.5 × 10^4^ cells/well in culture tubes and incubated at 37 °C in a carbon dioxide incubator for 24 h until confluence for inoculation was achieved.

The next day, each nasopharyngeal and salivary swab specimen was diluted at a ratio of 1:10 and inoculated into four wells containing EMEM (2% FBS, 1% P/S). Cells in the media were fixed with 10% formaldehyde and stained with 1% crystal violet dye.

The virus-induced cytopathic effect was examined daily for up to seven days [12,13].

## 3. Results

The analysis was performed on asymptomatic or mildly symptomatic COVID-19 patients who had been diagnosed and admitted to a CTC at least two weeks previously. In total, 48 patients were enrolled in this study. The mean age of all patients was 32.62 ± 14.59 years, and 14 of the patients were male (29.2%). Of all the patients, 11 (22.9%) were asymptomatic.

Baseline characteristics were similar between the symptomatic and asymptomatic patient groups. There was no statistically significant difference in terms of age, sex, comorbidities, and symptoms. The most common symptoms were myalgia (32.4%), fever (29.7%), and headache (24.3%); chest pain (2.7%) and vomiting (2.7%) were uncommon (Table 1).

Nasopharyngeal and salivary swab specimens were collected from the patients approximately 30.40 ± 5.71 days after initial diagnosis. The average Ct value of patients on the day of culture was over 30 in both the symptomatic and asymptomatic groups. A total of 17 cases (35.4%) showed rebound Ct values, which included 11 cases in the symptomatic group and 6 in the asymptomatic group (Table 2).

RT-PCR assay was performed using saliva specimens to determine the effectiveness of saliva as a diagnostic tool. As shown in Table 3, in the case of Patient 8, the saliva RT-PCR result was positive, although the RT-PCR result of the nasopharyngeal swab specimen was negative. Additionally, in eight patients, the mean Ct values of the nasopharyngeal and salivary swab specimens were 33.7 and 33.96, respectively, indicating that saliva swabs can serve as a reliable tool for diagnosing SARS-CoV-2 infection.

**Figure 1:**
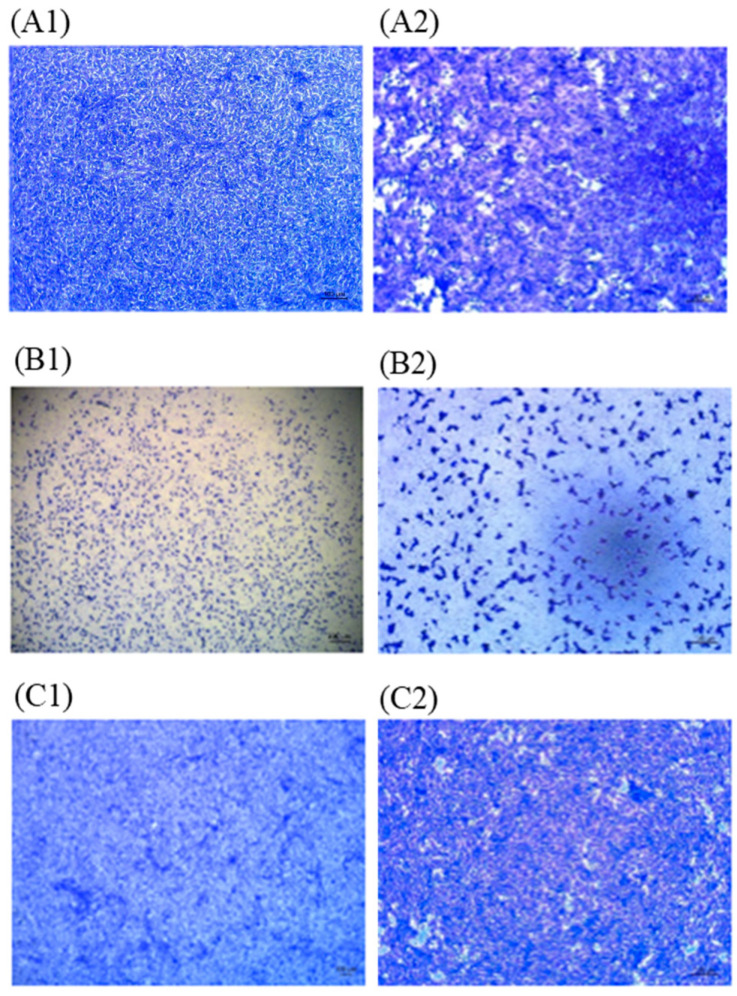
Microscopy image of vero monolayers. Vero cells were inoculated with nasopharyngeal swab. (A) Vero cell cultures in negative control. 40× (**A1**), 200× (**A2**) micrograph. (B) Vero cell cultures in positive control. 40× (**B1**), 200× (**B2**) micrograph. (C) Microscopy image of Vero cells inoculated with nasopharyngeal swab sample of a patient 33 days after diagnosis. No cytopathic effect was seen. 40× (**C1**), 200× (**C2**) micrograph. Virus stock used for positive control was obtained from the Centers for Disease Control and Prevention (KCDC, Seoul, Korea), the virus titer was 2.2 × 10^5^ PFU/mL and the Ct value was 11 (KCDC03 43326).

**Figure 2:**
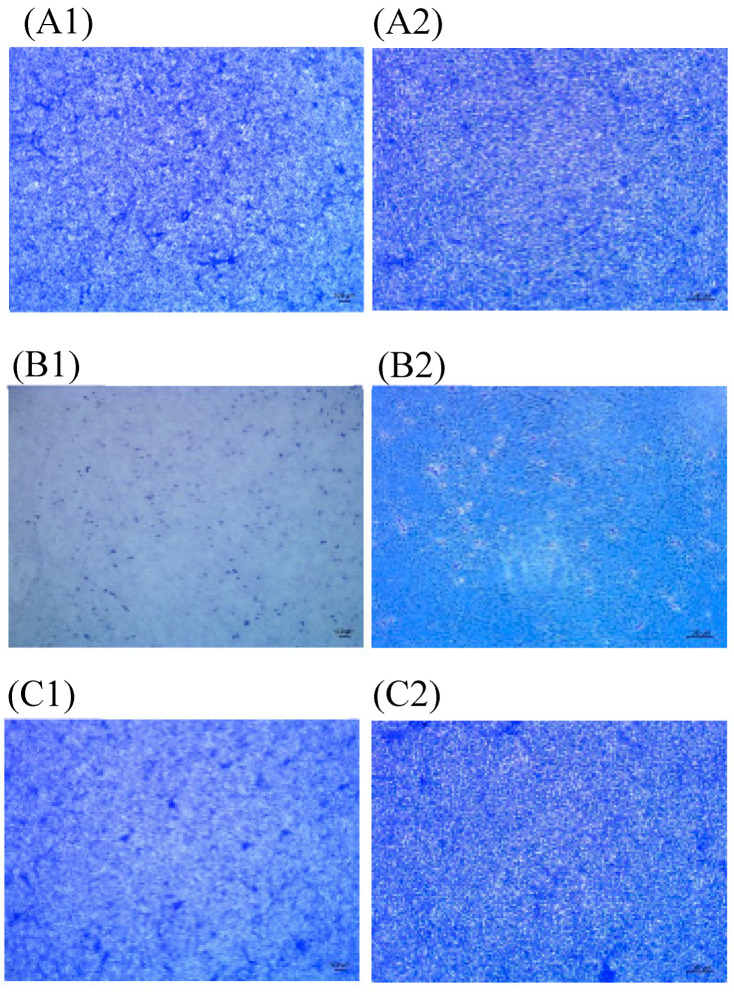
Microscopy image of vero monolayers. Vero cells were inoculated with salivary swab. (A) Vero cell cultures in negative control. 40× (**A1**), 100× (**A2**) micrograph. (B) Vero cell cultures in positive control. 40× (**B1**), 100× (**B2**) micrograph. (C) Microscopy image of Vero cells inoculated with salivary swab sample of a patient 33 days after diagnosis. No cytopathic effect was seen. 40× (**C1**), 100× (**C2**) micrograph. Virus stock used for positive control was obtained from the Centers for Disease Control and Prevention (KCDC), the virus titer was 2.2 × 10^5^ PFU/mL and the Ct value was 11. (KCDC03 43326).

Cell culture was performed using nasopharyngeal and salivary swab specimens to confirm the isolation of viable virus. Vero cells were inoculated with nasopharyngeal and salivary swab specimens and microplates were observed for the evidence of cytopathic effect. Specimens were collected from patients at least 20 days after diagnosis, and we found no microplates showing any cytopathic effect with these specimens (Figure 1 and Figure 2).

## 4. Discussion

In this study, we aimed to determine whether infectious viruses could be isolated using salivary and nasopharyngeal swab samples from patients with persistent positive RT-PCR results or rebound Ct values more than two weeks after diagnosis. SARS-CoV-2 could not be cultured from the patient specimens. Consequently, we surmised that there is no viral transmission risk in the later phase of SARS-CoV-2 infection. In addition, as indicated by the fact that the Ct values derived from salivary swab RT-PCR were similar to those of nasopharyngeal swab RT-PCR, saliva was shown to be an effective diagnostic tool for the detection of SARS-CoV-2.

SARS-CoV-2 infection is confirmed by detecting the presence of viral RNA through molecular testing, usually by RT-PCR [14]. The presence of viral RNA alone does not indicate that a patient is infectious or can transmit the virus to others. Factors affecting the transmission risk include whether the patient has symptoms, such as cough, which can spread droplets, whether a virus is infectious, and environmental factors or the behavior of the infected patient. Usually, 5 to 10 days after the initial SARS-CoV-2 infection, the patient begins to produce neutralizing antibodies. The binding of these neutralizing antibodies to the virus is expected to decrease the risk of viral transmission [4,15].

In many studies, viral shedding detected by RT-PCR from respiratory specimens has been found to persist for more than 20 days and sometimes up to 63 days after the onset of symptoms, and it appears to last beyond symptom resolution [6,8,16,17]. It has also been proven that transmission occurs in asymptomatic patients. In a study conducted by Zou et al., the viral loads of nasal and throat swabs were similar in symptomatic and permanently asymptomatic patients [18]. In addition, in the present study, the Ct values of symptomatic and asymptomatic patients were not significantly different. This might be because in the late phase of infection the viral load is close to the detection limit, but there is evidence that viral shedding occurs in both symptomatic and asymptomatic patients after symptom improvement; however, the relationship between the detection of viral RNA and infectivity is still unclear. RT-PCR results do not necessarily indicate the possibility of viral transmission and cannot distinguish between infectious and non-infectious virus [7,15].

Viral RNA has been detected in the upper and lower respiratory tract, blood, pharyngeal swabs, saliva, urine, and feces, regardless of the severity of the disease [5,16]. The virus has also been detected in water gargled by patients diagnosed with COVID-19 [17]. We used saliva specimens for RT-PCR tests to confirm SARS-CoV-2 infection. It was confirmed that saliva showed a high concordance rate of 90% or more with the nasopharyngeal specimens in the detection of respiratory viruses including coronavirus [13,19,20].

A study by Zhou et al. indicated that angiotensin-converting enzyme II (ACE2) is likely the cell receptor for SARS-CoV-2; it was also the receptor for SARS-CoV and HCoV-NL63 [4]. According to a previous study, ACE2 is presented on the epithelial cells of oral mucosa, suggesting that the oral cavity might be at high risk of SARS-CoV-2 infection [21]. These findings suggest that ACE2-expressing cells may act as target cells and are therefore vulnerable to SARS-CoV-2 infection [22]. Since many viruses, including SARS-CoV-2, can be detected in saliva, saliva is considered a major factor in the risk of transmission of viruses that can cause respiratory infections [21]. In our study, a viral load was detected in saliva, therefore, saliva can also be expected to serve as an effective diagnostic tool.

Viral RNA tends to be detected for a longer period of time in more severe cases. According to several studies, viral RNA has been detected in respiratory tract specimens 1-3 days before the onset of symptoms. Viral load is thought to increase for up to 7–10 days after the onset of symptoms in mild cases. In more severe cases, it tends to peak up to 11 days after the onset, followed by gradual decrease over time [4,23]. Some studies have predicted that transmission risk is the highest at or around the time of symptom onset and during the first five days of disease [16].

Viral culture studies using patient specimens to confirm the presence of infectious SARS-CoV-2 are still limited. In some studies, viable viruses have been isolated from viral cultures using respiratory samples collected during the early stages of the disease or at least within eight days after the onset of symptoms [4]. According to a study investigating the relationship between the Ct value and culture positivity rate, all samples with Ct values of 13-17 led to positive culture; however, the culture positivity rate decreased as Ct values increased. Cultures could not be obtained from specimens with Ct values ≥ 34 [24]. In the present study, the mean Ct value of specimens was approximately >30, and no virus was isolated in cultures using these samples.

This study has several limitations. Firstly, the sample size of this study was small and larger studies are needed to confirm the relationship between infectivity loss and RT-PCR results. Secondly, the infectivity of individual cases and accuracy of our culture analysis may have individualized variations. Furthermore, subculture is known to improve the sensitivity of culture assays, but subculture was not performed in this study. Fourth, since serial saliva specimens were not available, serial viral load monitoring was not possible in this study. Finally, changes in the dynamics of viral shedding could have occurred due to the treatments administered to patients.

Although viral RNA can be detected in RT-PCR analyses even after the improvement of symptoms, the amount of viral RNA gradually decreases over time, eventually reducing below the level at which viable virus can be isolated. Therefore, based on the evidence that the virus is rarely detected in respiratory specimens after 10 days following the onset of symptoms, especially in mild or asymptomatic cases of SARS-CoV-2 infection, even if viral shedding is sustained in the later phase of COVID-19, it can be expected that the transmission risk of the virus is low. Accordingly, it seems safer to release patients from quarantine based on the revised WHO discharge criteria that require minimum isolation for 13 days, than to repeat RT-PCR assays. Although the transmission risk is thought to be minimal in the later stages of COVID-19, it cannot be completely ruled out. Therefore, further investigations are warranted to understand the relationship between SARS-CoV-2 detection, viral culture, and transmission depending on the clinical course.

## Figures and Tables

**Table 1:** Comparisons of demographic and clinical characteristics of asymptomatic and symptomatic patients with COVID-19.

Variables	Total (*n* = 48)	Symptomatic Group (*n* = 37)	Asymptomatic Group (*n* = 11)	*p* Value
Age, years	32.62 ± 14.59	31.84 ± 13.74	35.27 ± 17.62	0.499
Male, yes	14 (29.2)	12 (32.4)	2 (18.2)	0.361
Smoker	5 (10.4)	5 (13.5)	0 (0.0)	0.198
Comorbidity				
Hypertension	4 (8.3)	2 (5.4)	2 (18.2)	0.178
Allergy	4 (8.3)	2 (5.4)	2 (18.2)	0.178
Malignancy	2 (4.2)	2 (5.4)	0 (0.0)	0.431
Diabetes	1 (2.1)	1 (2.7)	0 (0.0)	0.582
Asthma	1 (2.1)	1 (2.7)	0 (0.0)	0.582
Symptoms				
Myalgia	12 (25.0)	12 (32.4)	0 (0.0)	-
Fever	11 (22.9)	11 (29.7)	0 (0.0)	-
Chill	9 (18.8)	9 (24.3)	0 (0.0)	-
Headache	9 (18.3)	9 (24.3)	0 (0.0)	-
Anosmia	8 (16.7)	8 (21.6)	0 (0.0)	-
Ageusia	7 (14.6)	7 (18.9)	0 (0.0)	-
Rhinorrhea	7 (14.6)	7 (18.9)	0 (0.0)	-
Nasal stuffiness	7 (14.6)	7 (18.9)	0 (0.0)	-
Fatigue	7 (14.6)	7 (18.9)	0 (0.0)	-
Cough	6 (12.5)	6 (16.2)	0 (0.0)	-
Sputum	6 (12.5)	6 (16.2)	0 (0.0)	-
Sore throat	4 (8.3)	4 (10.8)	0 (0.0)	-
Dizziness	3 (6.3)	3 (8.1)	0 (0.0)	-
Diarrhea	3(6.3)	3 (8.1)	0 (0.0)	
Chest pain	1 (2.1)	1 (2.7)	0 (0.0)	-
Vomiting	1 (2.1)	1 (2.7)	0 (0.0)	-

COVID-19, coronavirus disease 2019; Continuous variables are shown as the mean ± standard deviation (SD) and categorical variables, as numbers (percentage).

**Table 2:** Virologic data of patients with COVID-19.

	Symptomatic Group (*n* = 37)	Asymptomatic Group (*n* = 11)	*p* Value
Period from diagnosis to culture			
Mean	30.78 ± 5.85	29.09 ± 5.26	0.394
Median	31 (20–40)	28 (21–41)	
Mean of Ct value on the day of culture			
*E* gene	30.55 ± 2.96	27.60 ± 0.47	-
*RdRP* gene	33.93 ± 3.43	31.56 ± 3.07	-
*N* gene	34.98 ± 2.89	33.94 ± 3.65	-
Cases with rebound of Ct value	11 (29.7)	6 (54.5)	0.131

Continuous variables are shown as the mean ± standard deviation (SD), median values (interquartile range), and categorical variables, as numbers (percentage). COVID-19, coronavirus disease 2019; Ct, cycle threshold; E, envelop protein; N, nucleocapsid protein; RdRP, RNA-dependent RNA polymerase; NPS, nasopharyngeal swab. Ct value presented in this table correspond to RT-PCR result of nasopharyngeal swabs.

**Table 3:** Clinical characteristics and Ct value of patients with positive salivary RT-PCR result.

Items	Age	Sex	Comorbidity	Symptoms	Rebound	Ct Value	
						NPS	Saliva
Patient1	51	M	N	Y	N	36.6	31.1
Patient2	21	M	Allergy	Y	N	38.9	37.0
Patient3	23	F	N	Y	N	35.1	31.4
Patient4	25	F	N	Y	N	32.2	35.2
Patient5	31	F	N	Y	N	33.2	36.9
Patient6	54	F	N	N	N	34.5	35.1
Patient7	27	F	N	Y	N	25.4	29.3
Patient8	33	M	N	Y	Y	0.0	35.7
Average of Ct value						33.7	33.96

RT-PCR, reverse transcriptase polymerase chain reaction; Ct, cycle threshold; NPS, Nasopharyngeal swab.

## References

[1] World Health Organization Coronavirus Disease (COVID-19) Situation Report—179. https://www.who.int/docs/default-source/coronaviruse/situation-reports/20200717-covid-19-sitrep-179.pdf?sfvrsn=2f1599fa_2.

[2] World Health Organization Criteria for Releasing COVID-19 Patients from Isolation. https://www.who.int/news-room/commentaries/detail/criteria-for-releasing-covid-19-patients-from-isolation.

[3] Azzi L., Carcano G., Gianfagna F., Grossi P., Gasperina D.D., Genoni A., Fasano M., Sessa F., Tettamanti L., Carnici F. (2020). Saliva is a reliable tool to detect SARS-CoV-2. J. Infect..

[4] Wolfel R., Corman V.M., Guggemos W., Seilmaier M., Zange S., Muller M.A., Niemeyer D., Jones T.C., Vollmar P., Rothe C. (2020). Virological assessment of hospitalized patients with COVID-2019. Nature.

[5] Peng L., Liu J., Xu W., Luo Q., Chen D., Lei Z., Huang Z., Li X., Deng K., Lin B. (2020). SARS-CoV-2 can be detected in urine, blood, anal swabs, and oropharyngeal swabs specimens. J. Med. Virol..

[6] To K.K.-W., Tsang O.T.-Y., Leung W.-S., Tam A.R., Wu T.-C., Lung D.C., Yip C.C.-Y., Cai J.-P., Chan J.M.-C., Chik T.S.-H. (2020). Temporal profiles of viral load in posterior oropharyngeal saliva samples and serum antibody responses during infection by SARS-CoV-2: An observational cohort study. Lancet Infect. Dis..

[7] Widders A., Broom A., Broom J. (2020). SARS-CoV-2: The viral shedding vs infectivity dilemma. Infect. Dis. Health.

[8] Zhou F., Yu T., Du R., Fan G., Liu Y., Liu Z., Xiang J., Wang Y., Song B., Gu X. (2020). Clinical course and risk factors for mortality of adult inpatients with COVID-19 in Wuhan, China: A retrospective cohort study. Lancet.

[9] Kim S.W., Lee K.S., Kim K., Lee J.J., Kim J.-Y. (2020). Daegu Medical Association a brief telephone severity scoring system and therapeutic living centers solved acute hospital-bed shortage during the COVID-19 outbreak in Daegu, Korea. J. Korean Med. Sci..

[10] Sung H., Roh K.H., Hong K.H., Seong M.-W., Ryoo N., Kim H.S., Lee J., Kim S.Y., Yoo S., Kim M.-N. (2020). COVID-19 Molecular testing in Korea: Practical essentials and answers from experts based on experiences of emergency use authorization assays. Ann. Lab. Med..

[11] Lai C.-C., Wang C.-Y., Ko W.-C., Hsueh P.-R. (2020). In vitro diagnostics of coronavirus disease 2019: Technologies and application. J. Microbiol. Immunol. Infect..

[12] Harcourt J., Tamin A., Lu X., Kamili S., Sakthivel S.K., Murray J., Queen K., Tao Y., Paden C.R., Zhang J. (2020). Isolation and characterization of SARS-CoV-2 from the first US COVID-19 patient. bioRxiv.

[13] To K.K.-W., Tsang O.T.-Y., Yip C.C.-Y., Chan K.-H., Wu T.-C., Chan J.M.-C., Leung W.-S., Chik T.S.-H., Choi C.Y.-C., Kandamby D.H. (2020). Consistent detection of 2019 novel coronavirus in saliva. Clin. Infect. Dis..

[14] Corman V.M., Landt O., Kaiser M., Molenkamp R., Meijer A., Chu D.K., Bleicker T., Brünink S., Schneider J., Schmidt M.L. (2020). Detection of 2019 novel coronavirus (2019-nCoV) by real-time RT-PCR. Eurosurveillance.

[15] Torres I., Sacoto F. (2020). Localising an asset-based COVID-19 response in Ecuador. Lancet.

[16] He X., Lau E.H.Y., Wu P., Deng X., Wang J., Hao X., Lau Y.C., Wong J.Y., Guan Y., Tan X. (2020). Temporal dynamics in viral shedding and transmissibility of COVID-19. Nat. Med..

[17] Liu W.-D., Chang S.-Y., Wang J.-T., Tsai M.-J., Hung C.-C., Hsu C.-L., Chang S.-C. (2020). Prolonged virus shedding even after seroconversion in a patient with COVID-19. J. Infect..

[18] Zou L., Ruan F., Huang M., Liang L., Huang H., Hong Z., Yu J., Kang M., Song Y., Xia J. (2020). SARS-CoV-2 viral load in upper respiratory specimens of infected patients. N. Engl. J. Med..

[19] To K.K.-W., Lu L., Yip C.C., Poon R.W., Fung A.M., Cheng A., Lui D.H., Ho D.T., Hung I.F.-N., Chan K.-H. (2017). Additional molecular testing of saliva specimens improves the detection of respiratory viruses. Emerg. Microbes Infect..

[20] To K.K.-W., Yip C.C., Lai C.Y., Wong C.K., Ho D.T., Pang P.K.P., Ng A.C., Leung K.-H., Poon R.W., Chan K.-H. (2019). Saliva as a diagnostic specimen for testing respiratory virus by a point-of-care molecular assay: A diagnostic validity study. Clin. Microbiol. Infect..

[21] Li Y., Ren B., Peng X., Hu T., Li J., Gong T., Tang B., Xu X., Zhou X. (2020). Saliva is a non-negligible factor in the spread of COVID-19. Mol. Oral Microbiol..

[22] Xu H., Zhong L., Deng J., Peng J., Dan H., Zeng X., Li T., Chen Q. (2020). High expression of ACE2 receptor of 2019-nCoV on the epithelial cells of oral mucosa. Int. J. Oral Sci..

[23] Pan X., Chen D., Xia Y., Wu X., Li T., Ou X., Zhou L., Liu J. (2020). Asymptomatic cases in a family cluster with SARS-CoV-2 infection. Lancet Infect. Dis..

[24] La Scola B., Le Bideau M., Andreani J., Hoang V.T., Grimaldier C., Colson P., Gautret P., Raoult D. (2020). Viral RNA load as determined by cell culture as a management tool for discharge of SARS-CoV-2 patients from infectious disease wards. Eur. J. Clin. Microbiol. Infect. Dis..

